# Evidence for Peripheral Immune Activation in Parkinson’s Disease

**DOI:** 10.3389/fnagi.2021.617370

**Published:** 2021-04-30

**Authors:** Xueping Chen, Weihua Feng, Ruwei Ou, Jiao Liu, Jing Yang, Jiajia Fu, Bei Cao, Yongping Chen, Qianqian Wei, Huifang Shang

**Affiliations:** ^1^Department of Neurology, West China Hospital, Sichuan University, Chengdu, China; ^2^Division of Clinical Immunology, West China Hospital, Sichuan University, Chengdu, China

**Keywords:** humoral immune, cellular immune, Parkinson’s disease, disease process, peripheral immunity

## Abstract

**Background:**

Accumulating evidence has revealed that peripheral immunity is involved in Parkinson’s disease (PD). However, the results regarding the percentage of T-cell subsets are inconsistent, and the changes of immunoglobins levels have been seldom studied in PD patients.

**Methods:**

Serum levels of the percentage of T-cell subsets and immunoglobulins were measured in 761 PD patients and 761 age- and gender-matched healthy controls. The correlations between the variables of peripheral immune activation (PIA) and the clinical characteristics of PD were analyzed using correlation analysis.

**Results:**

The pooled results showed that PD patients had higher proportional levels of CD3+ T and CD4+ T lymphocytes than healthy controls. CD8+ T cell percentages were similar in PD patients and controls, and the CD4/CD8 ratio was significantly higher in the PD population. No significant differences in IgG, IgA, or IgM levels between these two groups were found. CD4+ T cell percentage was inversely correlated with the H&Y stage, and IgG level was positively correlated with disease duration and UPDRS part III. Subgroup analyses showed that these associations existed in female patients, but not in male patients.

**Conclusion:**

The enhanced immune activation in the peripheral system is indicated in PD, and dynamic alterations in CD4+ T cell percentage and IgG level suggest an active role for peripheral immunity in the disease progression, especially in female PD patients.

## Introduction

Parkinson’s disease (PD) is a progressive neurodegenerative disorder, and the role of adaptive immune system and humoral immunity needs to be clarified in PD; profiling of peripheral T cells and immunoglobulin may be able to provide insight into biologic markers of disease. Adaptive immunity has become implicated as a pathogenic factor in the onset and progression of PD. The infiltration of immune cells in the central nervous system (CNS), particularly the substantia nigra (SN), was identified in both postmortem PD patients and animal models (Reynolds et al., 2007; Tufekci et al., 2011). These brain-invading immune cells consisted of a heterogeneous population of both CD8+ and CD4+ T cells. Studies had found a significant increase in the density of CD8+ and CD4+ T cells in the SN of PD patients compared with age-matched control subjects (Brochard et al., 2009). Active T cells are detected not only in the CNS, but also in the peripheral blood (Rodrigues et al., 2014). Both clinical study and experimental model exhibit blood-brain barrier (BBB) dysfunction and T lymphocytes’ recruitment to the CNS in PD. Besides direct function at the inflammatory site, the peripheral immune cells may get involved in the pathogenesis of PD systemically. Interestingly, CNS is reported to be actively interacts with the peripheral immune system (Aloisi, 2001; Wirenfeldt et al., 2011). Previous studies reported that PD patients with stage from I to III, or stage from I to V evaluated using the Hoehn and Yahr (H&Y) scale, displayed an altered population of peripheral T-lymphocyte than age-matched control subjects (Hisanaga et al., 2001; Baba et al., 2005). Studies have shown the involvement of peripheral adaptive immunity in PD (Kustrimovic et al., 2018; De Francesco et al., 2021), and the decrease of overall numbers of lymphocytes in PD patients (Bas et al., 2001; Calopa et al., 2010). Moreover, the peripheral immune change in PD patients was evidenced by decreased percentage of CD3+ T lymphocytes and CD4+ T lymphocytes in peripheral blood (Cen et al., 2017; Rocha et al., 2018; Sun et al., 2019). The reduced circulating CD4+ T lymphocytes in PD patients were shown to be caused by the reduction in Th2, Th17, and Treg (Kustrimovic et al., 2018), and the reduction of CD4+ T cells was worsened with increasing clinical severity (Stevens et al., 2012). A meta-analysis included 943 PD patients and found that the numbers of CD3+ and CD4+ T lymphocyte subsets were decreased in PD patients (Jiang et al., 2017). PD patients were also found to have a lower CD4+:CD8+ ratio than age-matched controls (Bas et al., 2001; Baba et al., 2005). However, another study did not find any difference in the percentage of CD4+ or CD8+ T lymphocytes and the CD4+:CD8+ ratio between PD patients and controls (Rocha et al., 2018). Furthermore, some studies found that PD patients showed higher levels of CD4+ or CD8+ T lymphocytes in peripheral blood (Garfias et al., 2019). Therefore, the results of the composition of T cells in the blood of PD patients are not consistent (Jiang et al., 2017); probably due to the relatively small sample size and ethnic variations. In addition, a Meta-analysis included 21 case-control studies, and the sample size of these studies varied from 20 to 82. Among these studies 16 were from the Asian population, and five were from the Caucasian population; a significant heterogeneity was observed in the analysis of T lymphocytes, and one reason of heterogeneity may be ascribed to ethnic variations (Jiang et al., 2017). The present study will focus on the changes in the percentage of T-cell subsets in PD patients relative to age/gender-matched healthy controls, trying to shed some light on whether adaptive immune responses are altered in PD.

Given the chronic inflammatory nature of PD, humoral immunity could play another role in disease progression. Lewy bodies (LB) shows strong immunolabelling for immunoglobulin G (IgG) and about one-third of DA neurons in SN show surface immunoreactivity for IgG (Sanchez-Guajardo et al., 2013), and the surface coating of SN DA neurons with IgG may target them for early degradation in the disease process (Orr et al., 2005). In the mouse model, the AAV-induced intra-nigral expression of human wild-type α-synuclein leads to significant IgG deposition, indicating that humoral immunity is involved in the degenerative process (Theodore et al., 2008). Humoral immunity and the secreted immunoglobulins can affect at sites far from the actual location of secretion, antibodies against α-synuclein have been found in sera and cerebrospinal fluid (CSF) of PD patients (Papachroni et al., 2007). Another study showed that sera from PD patients contained disease-specific auto-antibodies, because characteristic alterations of 10 auto-antibodies could distinguish PD from normal aging, Alzheimer’s disease (AD), multiple sclerosis, and breast cancer with accuracies over 85%, and these 10 auto-antitbodies included PTCD2, HSH2D, Myotilin, Elongation factor 1-alpha 1, ICAM4, FRMD8, CTLA-4/Fc, PABPC3, Fibronectin 1, and TRIM21 (Han et al., 2012).

Yet another study found that the frequency of anti-synuclein antibodies in the peripheral blood serum of sporadic PD patients is not different from those in healthy controls (Papachroni et al., 2007). This is the first study that focusing on the change of serum immunoglobulins relative to age/gender-matched healthy controls, trying to provide more insight into the role of immunoglobulins in PD pathogenesis.

Since functional profiling of peripheral immune activities could provide important disease biomarkers, we conducted this study to investigate peripheral immune abnormalities in PD. The current study was designed: (1) to measure the percentage of T cell subset, and the levels of serum immunoglobulins IgG, IgM, IgA in PD patients and normal controls and (2) to test whether the peripheral immune activation (PIA) state of PD patients would correlate with disease characteristics.

## Materials and Methods

### Patients and Healthy Individual

This study was performed within the Department of Neurology, West China Hospital of Sichuan University; a large, regional referral movement disorders clinic in Southwest China. A total of 761 PD patients who met the current consensus criteria for PD (Postuma et al., 2015) were recruited and followed from May 2006 to November 2018. A total of 761 Age- and gender-matched healthy controls were recruited from the medical examination center of West China Hospital; the control subjects did not have any neurological conditions or systemic diseases, such as hypertension and diabetes. All patients and control subjects gave their written informed consent, and the Ethical Committee of West China Hospital of Sichuan University approved this study.

### Exclusion Criteria

Patients who showed evidence of systemic inflammation in clinical examination or biochemical blood tests (increased number of white blood cells, high concentration of C-reactive protein, or high erythrocyte sedimentation rate) were excluded. Patients with manifestations of monoclonal gammopathy, non-malignant endocrine abnormalities, neoplastic disorders, auto-antibodies (high-titer GM1 ganglioside antibody), and infection (HIV-1, hepatitis B and C, varicella-zoster, syphilis, borreliosis) were also excluded. None of the patients had a history of using anti-inflammatory drugs, steroids, acetylsalicylic acid or statins during the past 2 months before enrollment. Any patient with a family history of PD or common chronic diseases was also excluded.

### Collection and Processing of Blood Specimens

In accordance with study protocols, peripheral blood samples (∼9 ml each set) were obtained by venipuncture performed between 9:00 and 11: 00 am after fasting from midnight from each patient and healthy controls. Blood was collected into a sterile 7.5 ml tube with a clot activator and double gel for transport (BD Vacutainer, SST, REF 367987). All the experiments in this study were conducted in the Division of Clinical Immunology, West China Hospital, affiliated with Sichuan University. Flow cytometric assay for analysis of cell viability was performed. The lymphocyte subsets were identified and determined using the BD FACSCanto II Flow Cytometer [Becton Dickinson (BD) Biosciences, Heidelberg, Germany]. For each sample, at least 10,000 cells were analyzed and the percentage of the cells expressing CD3+, CD4+ and CD8+ markers were evaluated. The levels of immunoglobulins IgG, IgM, and IgA were quantified by means of nephelometry on an Image 800 nephelometer (Beckman Coulter, Fullerton, CA, United States). A portion of each collected sample was analyzed to detect infectious diseases. According to the manufacturer’s instructions, all the tests were performed by board-certified laboratory technicians who were blinded to clinical data.

### Clinical Evaluation

We collected clinical data regarding sex, age at onset, disease duration, diagnostic delay, personal history, chronic disease history, treatment regimen and motor complications through a standardized personal interview. The diagnostic delay is defined as the duration from the first appearance of motor symptoms to clinical diagnosis of PD. We made a detailed record of chronic disease history, such as hypertension, diabetes, and hyperlipemia. The use of antiparkinson drug was recorded at the time of enrollment and L-DOPA equivalent daily doses (LEDD) were evaluated according to the guidelines (Tomlinson et al., 2010). Patients who reported one or more of the following symptoms, including wearing-off, delayed-ON, no-ON, random ON-OFF, early morning/nocturnal akinesia, peak-dose or diphasic dyskinesias, were judged to have motor complications. The Unified PD Rating Scale (UPDRS) part III and H&Y stage were used to evaluate the motor severity (Hoehn and Yahr, 1967; Movement Disorder Society Task Force on Rating Scales for Parkinson’s Disease, 2003). The severity of non-motor symptoms (NMSs) was assessed with the Non-Motor Symptom Scale (NMSS) (Chaudhuri et al., 2007). The Chinese version of the NMSS includes nine domains and 30 items, and it is a valuable measure to assess the frequency and severity of NMS in Chinese PD patients (Wang et al., 2009). The quality of life (QoL) of PD patients was evaluated with the Chinese version of the PD Questionnaire 39 (PDQ-39) (Jenkinson et al., 1997).

### Statistical Analysis

The distribution of the detected variables of PIA, including percentage of T lymphocyte subset and levels of IgG, IgM, and IgA were tested for normality using skewness and kurtosis, Shapiro–Wilk and Shapiro–Francia tests. The Student’s *T*-test was applied to analyze the difference in the detected variables of PIA for comparisons between PD patients and controls. Chi-square tests compared sex ratios between PD and control groups. Spearman’s rho correlation was used to calculate the correlations between the detected PIA variables and the H&Y stage, while Pearson’s correlation was applied to evaluate the correlations with UPDRS part III, NMSS, and PDQ-39. Multivariate general linear models (GLMs) were used to investigate the associations with clinical variables, with CD4+ T lymphocyte percentage or IgG level as an independent variable. Clinical variables included in the GLMs were as follows: age at assessment, sex, disease duration, UPDRS part III, and LEDD. All data was presented in the form of mean ± standard deviation, and they were analyzed using SPSS 17.0. A *p*-value of < 0.05 was considered statistically significant.

## Results

The demographic features of included PD patients and controls were listed in Table 1. In the 761 PD patients, the sex ratio (male/female) was 395/366; the mean age at examination was 61.77 ± 11.19 years, which were not significantly different from controls. The results of the detected variables of PIA and the demographic data in PD patients were summarized in Table 1. Analysis of the T cell subset showed that PD patients had significantly higher proportional levels of CD3+ T and CD4+ T lymphocytes than healthy controls. CD8+ T cell percentages were similar in PD patients and controls. Consequently, the CD4/CD8 ratio was significantly higher in the PD population (Table 1). The T cell subset was evaluated separately according to gender. In both PD patients and controls, increased percentages of CD3+ T and CD4+ T lymphocyte populations were found in females (Table 2). The differences in age at assessment, disease duration, UPDRS part III, H&Y stage, NMSS, PDQ-39, and LEDD between female PD patients and male PD patients were evaluated, and the results showed that the differences in these variables were not significant (Table 2). We further examined the correlations between T cell subset and clinical data, including disease duration, UPDRS part III, H&Y stage, NMSS, LEDD, and PDQ-39. The results showed that CD4+ T cell percentage in PD patients was inversely correlated with the H&Y stage (*p* = 0.024) (Table 3). PD patients in a more advanced stage had a lower CD4+ T cell percentage. However, we did not find a significant association between CD4+ T cell percentage and sex, disease duration, UPDRS part III, and LEDD in GLMs. Still, a relationship between CD4+ T cell percentage and age at assessment was identified (*p* = 0.002). In the subgroup analyses of patients with different gender, CD4+ T cell percentage was found to be inversely correlated with UPDRS part III, H&Y stage, LEDD, and PDQ-39 in female patients, but not in male patients (Tables 4, 5). Female patients with a lower CD4+ T cell percentage were more likely to experience more severe motor symptoms and worse quality of life. In addition, the subgroup analyses using GLMs also showed that CD4+ T cell percentage was correlated to age at assessment in female patients (*p* = 0.003).

**TABLE 1:** Comparison of peripheral immune variables in PD patients and matched controls.

	PD patients n = 761	Controls n = 761	*p-value*
Age-of-onset	61.77 ± 11.19	61.67 ± 11.16	0.8670
Male/female	395/366	395/366	1
UPDRS part III	33.29 ± 15.12		
H&Y stage	2.44 ± 0.75		
NMSS	46.22 ± 36.00		
LEDD	376.52 ± 315.53		
PDQ-39	40.00 ± 27.80		
CD3%	63.36 ± 10.90	59.75 ± 12.53	**< 0.0001**
CD4%	36.50 ± 8.95	32.17 ± 8.83	**< 0.0001**
CD8%	22.57± 8.41	22.77± 8.93	0.6562
CD4/CD8	1.95± 1.75	1.64± 0.82	**< 0.0001**
IgG	12.25± 3.09	12.51± 3.60	0.0962
IgM	1227± 1305	1173± 745.9	0.2713
IgA	2212± 1065	2309± 1316	0.0798

*Bold values mean that the difference is statistically significant.*

**TABLE 2:** Comparison of peripheral immune variables according to gender of PD patients and matched controls.

Variables	PD patients			Controls		
	Male	Female	*p-value*	Male	Female	*p-value*
CD3%	62.28 ± 11.16	64.68 ± 10.36	**0.0022**	58.02 ± 12.96	61.63 ± 11.79	**<0.0001**
CD4%	35.42 ± 9.18	37.71 ± 8.49	**0.0004**	30.89 ± 9.27	33.56 ± 8.10	**<0.0001**
CD8%	22.50 ± 8.57	22.67 ± 8.64	0.7903	22.63 ± 9.26	22.91 ± 8.56	0.6681
CD4/CD8	1.96 ± 2.26	1.93 ± 0.95	0.8364	1.61 ± 0.86	1.68 ± 0.77	0.2316
IgG	12.21 ± 3.42	12.31 ± 2.61	0.6411	12.29 ± 3.93	12.80 ± 3.10	**0.0299**
IgM	1155 ± 1638	1317 ± 685.4	0.0587	1079 ± 746.5	1292 ± 728.6	**<0.0001**
IgA	2218 ± 1163	2205 ± 928.1	0.8494	2167 ± 2355	2220 ± 2518	0.2111

*Bold values mean that the difference is statistically significant.*

**TABLE 3:** Clinical characteristics of PD patients and the associations with T cell subsets and immunoglobulins levels.

Variables	CD3%		CD4%		CD8%		CD4/CD8		IgG		IgM		IgA	
	*r*	*p*-value	r	*p*-value	*r*	*p*-value	*r*	*p*-value	*r*	*p*-value	*r*	*p*-value	*r*	*p*-value
Disease duration	−0.073	0.175	−0.033	0.542	−0.029	0.586	0.050	0.360	**0.036**	**0.011**	−0.068	0.206	−0.001	0.990
UPDRS part III	−0.063	0.240	0.097	0.074	0.047	0.385	−0.019	0.719	**0.117**	**0.035**	−0.085	0.116	−0.035	0.514
H&Y stage	−0.099	0.067	**−0.121**	**0.024**	0.033	0.539	0.068	0.207	0.065	0.226	−0.079	0.146	−0.018	0.743
NMSS	−0.021	0.703	−0.037	0.496	0.074	0.170	−0.059	0.273	0.078	0.147	−0.063	0.215	0.063	0.242
LEDD	−0.064	0.236	−0.060	0.762	−0.016	0.762	0.020	0.715	−0.004	0.941	−0.099	0.067	−0.046	0.392
PDQ-39 total	−0.046	0.406	−0.037	0.509	0.019	0.738	−0.014	0.806	0.057	0.308	−0.92	0.095	−0.090	0.095

*Bold values mean that the difference is statistically significant.*

**TABLE 4:** Clinical characteristics of female PD patients and the associations with T cell subsets and immunoglobulins levels.

Variables	CD3%		CD4%		CD8%		CD4/CD8		IgG		IgM		IgA	
	*r*	*p*-value	r	*p*-value	r	*p*-value	*r*	*p*-value	r	*p*-value	*r*	*p*-value	*r*	*p*-value
Disease duration	−0.108	0.178	−0.104	0.192	0.010	0.896	0.020	0.801	**0.173**	**0.029**	−0.082	0.303	−0.028	0.726
UPDRS part III	−0.087	0.278	**−0.165**	**0.038**	0.073	0.361	−0.067	0.406	0.113	0.158	0.054	0.500	−0.016	0.843
H&Y stage	−0.139	0.082	**−0.201**	**0.011**	0.039	0.627	−0.106	0.183	0.039	0.630	−0.123	0.124	0.037	0.644
NMSS	−0.064	0.426	−0.093	0.246	0.043	−0.074	0.354	0.273	0.019	0.808	−0.143	0.072	0.072	0.371
LEDD	−0.144	0.072	**−0.172**	**0.031**	0.019	0.809	−0.075	0.347	0.017	0.827	−0.085	0.287	−0.084	0.294
PDQ-39 total	0.075	0.351	**−0.194**	**0.017**	−0.046	0.575	−0.022	0.789	0.086	0.293	−0.153	0.061	−0.067	0.402

*Bold values mean that the difference is statistically significant.*

**TABLE 5:** Clinical characteristics of male PD patients and the associations with T cell subsets and immunoglobulins levels.

Variables	CD3%		CD4%		CD8%		CD4/CD8		IgG		IgM		IgA	
	*r*	*p*-value	*r*	*p*-value	*r*	*p*-value	*r*	*p*-value	*r*	*p*-value	*r*	*p*-value	*r*	*p*-value
Disease duration	0.052	0.482	0.019	0.797	−0.069	0.349	0.077	0.299	0.105	0.153	−0.066	0.369	0.026	0.726
UPDRS part III	−0.030	0.680	−0.038	0.604	0.031	0.675	0.017	0.819	0.117	0.113	−0.045	0.093	−0.045	0.540
H&Y stage	−0.063	0.394	0.049	0.507	0.023	0.753	−0.029	0.692	0.092	0.210	−0.043	0.562	−0.065	0.381
NMSS	0.006	0.940	−0.001	0.992	0.100	0.177	−0.047	0.524	0.137	0.061	−0.006	0.936	0.046	0.529
LEDD	0.003	0.969	0.024	0.744	−0.046	0.530	0.098	0.185	−0.023	0.752	−0.111	0.131	−0.005	0.946
PDQ-39 total	0.056	0.459	0.066	0.378	0.069	0.359	−0.009	0.904	0.037	0.626	−0.065	0.392	0.089	0.239

The levels of immunoglobulins IgG, IgM, and IgA were determined by routine laboratory testing. The results showed no significant differences in IgG, IgM, and IgA level between PD patients and controls (Table 1). The subgroup analysis by gender revealed that the levels of immunoglobulins IgG, IgM, and IgA were not significantly different between male and female PD patients (Table 2). Furthermore, IgG level was positively correlated with disease duration and UPDRS part III (*p* = 0.011 and 0.031, respectively) (Table 3). Higher IgG level was associated with longer disease duration and more severe motor symptoms. Furthermore, the results of GLMs showed that IgG level was significant correlated with disease duration (*p* = 0.011). In subgroup analyses, a positive relationship between IgG level and disease duration was found in female PD patients (Tables 4, 5), and GLMs also found a positive association between IgG level and disease duration in female PD patients (*p* = 0.049).

## Discussion

Recent studies have indicated that the activity of the immune system in the CNS of PD patients, however; few studies to date have explored the status of peripheral immune response. To our knowledge, the current study included the largest sample in determining whether peripheral immune abnormalities are involved in PD by evaluating the T cell subsets and determining the humoral immunity in PD patients and healthy subjects.

In this study, we found that compared to healthy controls, PD patients had an increase in the percentage of CD3+ and CD4+ T lymphocyte, no change in CD8+ T cell subpopulation, and consequently an increase in the CD4+/CD8+ ratio. Considering the T lymphocyte subsets, CD3+ T cells represent the total lymphocytes, CD4+ T cells represent the helper T cells and are associated with anti-inflammation, but CD8+ T cells represent the cytotoxic T cells and are related with pro-inflammation, and a declining of CD4+/CD8+ ratio indicates an impaired immune system (Luz Correa et al., 2014). Our findings suggested the enhanced immune activation in the peripheral system. Furthermore, persistently activated peripheral immune system were also observed in amyotrophic lateral sclerosis (ALS), multiple system atrophy (MSA), and AD (Shalit et al., 1995; Chen et al., 2014; Chen et al., 2016; Kempuraj et al., 2016; Cao et al., 2020). However, previous studies reported a reduction in the number of CD4+ cells in PD patients (Bas et al., 2001; Baba et al., 2005; Niwa et al., 2012), and a meta-analysis also showed the decreased numbers of CD3+ and CD4+ lymphocyte subsets in PD patients (Jiang et al., 2017). The present study was focusing the percentage of the T cell subset, which could not represent the numbers of T cells. A study identified a significant decrease in the number of CD4+ lymphocytes in PD patients, but did not find any significant difference in the percentage of CD4+ cells between PD patients and healthy controls (Kustrimovic et al., 2016). Previous studies found an ongoing loss of CD4+ T cells as the disease progresses in PD patients (Stevens et al., 2012), and PD patients with cognitive impairment had a higher number of circulating lymphocytes than patients with normal cognitive function (Magistrelli et al., 2020), and the heterogeneity of the PD patients may account for the discrepancies. In the present study, we found an increase in the percentage of CD4+ T lymphocyte in PD patients. Similarly, previous studies have found a slightly increased proportion of CD4+ T cells in AD patients (Shalit et al., 1995; Richartz-Salzburger et al., 2007). Systemic CD4+ T cells must be recruited to the CNS to modify potentially destructive neuro-inflammation, and the increase of CD4+ T cells proportion may represent the compensatory mechanism. Furthermore, dopaminergic antiparkinson drugs may have had a role in regulating the populations of lymphocytes in PD. An *in vivo* study found that levodopa treatment could inhibit the number of peripheral IFN-γ-producing T cells (Carr et al., 2003). In addition, a recent study found that the percentage of CD3+ T cells was higher, but the percentage of CD4+ T cells was lower in PD patients treated with antiparkinson drugs than drug naïve (Kustrimovic et al., 2016). However, another study reported that PD patients with or without levodopa treatment had similarly decreased lymphocyte counts (Bas et al., 2001). In the present study, we only found an association between the percentage of CD4+ T cells and LEDD; a higher dose of antiparkinson drugs was related to lower percentage of CD4+ T cells. Therefore, the influence of antiparkinsonian treatment on peripheral immunity should be assessed in future study.

According to the theory of Braak’s staging in PD, the pathogenesis is indicated to develop from the peripheral system to the CNS (Braak et al., 1996), and peripheral immune activation has been suggested to play a role in the etiology of PD. The infiltration of T cells in the brain has been reported in both postmortem PD patients and animal models, and these brain-invading lymphocytes are composed of a heterogeneous population of both CD4+ and CD8+ cells (Farkas et al., 2000; Brochard et al., 2009; Tufekci et al., 2011). Furthermore, the brain parenchyma migration of peripheral leukocytes is found to be tightly regulated at the level of the BBB (Alvarez et al., 2011), and breakdown of the BBB was indicated in multiple pathways of neurodegeneration, including AD, PD, and MSA (Sweeney et al., 2018). In the present study, CD4+ T cell percentage was found to be inversely correlated with the H&Y stage. Previous study have found that the numbers of CD4+ T cells were negatively correlated with UPDRS and H&Y stage (Stevens et al., 2012). As the disease progressed to an advanced stage, the CD4+ T cell subpopulation tends to be decreased; this reduction may reflect the attempt of the CNS to recruit CD4+ T lymphocytes from the periphery to modulate the brain immunological reaction. The infiltration of CD4+ T lymphocytes was indicated to be related to a decrease in the phagocytic activity of microglia (Sommer et al., 2016), and the specific involvement of the CD4+ T lymphocytes was demonstrated by the dopaminergic neuroprotection observed when: deleting CD4 cells in the MPTP mouse (Brochard et al., 2009) and the MHC-II global knockout mouse (Harms et al., 2013). However, we had to notice that the association between CD4+ T cell percentage and H&Y stage was relatively weak, and the results of GLMs did not support the findings. In addition, the inverse association between CD4+ T cell percentage and UPDRS part III, H&Y stage, and PDQ-39 was only identified in female PD patients. Increasing evidence points to gender differences as an important determinant of the susceptibility to develop PD and the clinical and therapeutic management of PD (Picillo et al., 2017; Cerri et al., 2019). Sex hormones, specifical estrogen, were shown to influence PD pathogenesis and play a vital role in PD differences between men and women (Jurado-Coronel et al., 2018). Estrogen can CD4+ T lymphocytes development and function, particularly their ability to produce selected cytokine profiles (Pernis, 2007).

In the present study, we only analyzed the percentage of T-cell subsets, but we had to notice that the transcription factor (TF) gene expression profile and the phenotypes of CD4+ T cells also represented the peripheral immune abnormalities in PD. For the TF gene expression, studies have shown that PD patients had higher levels of STAT6, GATA3, and FOXP3, and lower levels of TBX21, STAT3, STAT4, RORC, and NR4A2 than control subjects. In addition, patients with inidiopathic REM sleep behavior disorder (iRBD) were also had a similar TF gene expression with PD patients (De Francesco et al., 2021). Since iRBD was considered to be the strongest risk factor for prodromal PD, the altered TF gene expression in iRBD suggested early involvement of peripheral immunity in PD. Furthermore, PD patients with or without motor complications had different TF gene expression in CD4+ T cells; indicating the associations with disease progression (Contaldi et al., 2020). There are two phenotypes of CD4+ T cells, the proinflammatory phenotypes (Th1 and Th17), and the anti-inflammatory phenotypes (Th2 and the Treg). PD patients had a functional Th1-biased immune response in the peripheral immune system (Kustrimovic et al., 2018), and PD patients who had higher Th1 and a dysregulation of Treg tend to be more vulnerable to develop cognitive dementia (Magistrelli et al., 2020). Study also showed the associations of dopaminergic Receptors on Naïve CD4+ T cells and memory lymphocytes to motor impairment in PD patients (Kustrimovic et al., 2016). Combined with our findings, these studies’ results indicate that peripheral adaptive immunity is involved in PD.

In the present study, our study indicated that the serum levels of IgG, IgA, and IgM in PD patients did not differ from those in control subjects. Similarly, our previous study also found that IgG and IgM concentrations were not significantly different between ALS patients and healthy controls (Chen et al., 2014). We conducted a pilot study that showed no differences in serum immunoglobulin levels between ALS and PD patients (data not shown). Therefore, PD seems to share similar features with other neurodegenerative diseases with regard to peripheral immunity activation. However, a role for humoral immunity has also been proposed in PD. Immunoglobulins are found to colocalize with pigmented DA neurons, and LB in PD brains shows strong immunolabelling for IgG (Orr et al., 2005). A previous study injected immunoglobulins from PD patients into the SN of rats, and found that these immunoglobulins induced perivascular inflammation, microgliosis, and loss of TH+ neurons in the SN (Chen et al., 1998). Furthermore, DA neurons’ surface coating with IgG could target them for degradation early in PD progress (Orr et al., 2005). Researchers used AAV to induce intraneural expression of human wild type α-synuclein, and found a significant IgG deposition, indicating that humoral immunity and the BBB breakdown are involved in the pathogenesis of PD (Cao et al., 2010). Supporting this, we also found that IgG level was positively correlated with disease duration and UPDRS part III; as the disease progressed the increase of IgG may occur spontaneously with the deposition of aggregated α-synuclein and the DA neurons loss. Studies have shown that all humans have a great number of serum IgG auto-antibodies, but this number can be influenced by three factors: age, gender, and disease (Nagele et al., 2013). In the present study, we found a positive relationship between IgG level and disease duration in female patients, but further research is required to understand the influence of gender in systemic humoral immune responses in PD.

The principal role of neuroinflammation is indicated in the progression of PD, and the presence of activated microglia and the elevated cytokine levels in CNS are associated with neuroinflammation. Also, overstimulated immune system due to the gut dysbiosis and the enhanced intestinal permeability can persuade systemic inflammation in PD (Dogra et al., 2021). Therefore, the bidirectional communication between neuroinflammation and the peripheral immunity is involved in the pathobiology of PD (Kustrimovic et al., 2019).

Several limitations of this study need to be addressed. First, CD4+ T lymphocytes may be proinflammatory (Th 1 and Th17), or anti-inflammatory (Th2 and Treg). However, in the present study, we did not evaluate the imbalance of CD4+ T cell subsets. Second, a peculiar TF gene expression profile can reflect the imbalance of CD4+ T cells in PD. Therefore, it would be more convincing to examine the expression of peripheral TFs, including TBX21, STAT1, STAT3, STAT4, STAT6, RORC, GATA3, FOXP3, NR4A2, MEF2s, Nurr1, Pitx3, and En1/2. Third, we did not evaluate antiparkinsonian treatment’s effect on the periphery immunity in the present study.

## Conclusion

This study validated the enhanced immune activation in the peripheral system of PD. In addition, dynamic alterations in CD4+ T cell percentage and IgG level indicated an active role for peripheral immunity in the disease progression and neuronal health. A better understanding of the peripheral immune response is likely to have therapeutic implications; further prospective studies are required to confirm our findings.

## Data Availability Statement

The original contributions presented in the study are included in the article/supplementary material, further inquiries can be directed to the corresponding author/s.

## Ethics Statement

The studies involving human participants were reviewed and approved by The Ethical Committee of West China Hospital of Sichuan University. The patients/participants provided their written informed consent to participate in this study.

## Author Contributions

XC contributed the major role in the acquisition of data, analyzed the data, and drafted the manuscript for intellectual content. WF, RO, JL, JY, JF, BC, YC, and QW contributed to data collection. HS interpreted the data and revised the manuscript for intellectual content. All authors contributed to the article and approved the submitted version.

## Conflict of Interest

The authors declare that the research was conducted in the absence of any commercial or financial relationships that could be construed as a potential conflict of interest.
